# Trust as an Omnichannel-to-Advocacy Transmission Mechanism in Mandatory Health care Services: Empirical Evidence from Indonesia

**DOI:** 10.12688/f1000research.183317.2

**Published:** 2026-07-14

**Authors:** Erwin Suryanegara, Rhian Indradewa, Rina Anindita, Muhammad Gunawan Alif

**Affiliations:** 1 Management, Esa Unggul University, West Jakarta, Jakarta, Indonesia

**Keywords:** omnichannel integration; brand trust; brand advocacy; full mediation; credence service; mandatory healthcare; S-O-R Theory; PLS-SEM

## Abstract

**Background:**

In Indonesia, omnichannel integration in healthcare is intensifying, yet the psychological mechanisms that convert omnichannel integration into patient advocacy remain insufficiently understood, particularly in mandatory healthcare settings characterised by credence-service properties, high perceived risk, and constrained choice. This study uses the Stimulus-Organism-Response (S-O-R) framework to examine brand trust as a mediating mechanism linking omnichannel integration to brand advocacy.

**Methods:**

Data were collected from 230 patients across three health care facilities in Jakarta and Tangerang through questionnaires validated via forward translation, back translation, face validity, content validity, and pre-testing. Partial least squares structural equation modelling (PLS-SEM) with 5,000 bootstrap subsamples, supplemented by cross-loading evaluation, heterotrait-monotrait (HTMT) inference, model comparison, and demographic control variables, was used for analysis.

**Results:**

Omnichannel integration was positively associated with brand trust (β = 0.715; p < 0.001), and brand trust was positively associated with brand advocacy (β = 0.671; p < 0.001). However, the direct effect of omnichannel integration on brand advocacy was not significant (β = 0.019; p = 0.793). The indirect effect of omnichannel integration on brand advocacy through brand trust was significant (β = 0.480; 95% confidence interval [0.372, 0.586]), indicating a full mediation pattern (variance accounted for = 96.2%). After controlling for age, gender, and education, core relationships remained stable, and the structural model explained 46.8% of the variance in brand advocacy (R ^2^ = 0.468).

**Conclusions:**

These findings suggest that omnichannel integration is primarily associated with patient advocacy in mandatory health care services through trust. However, because this study does not directly compare service contexts, any inference regarding differences from conventional retail should be interpreted cautiously. As the data are cross-sectional, the reported relationships represent associations rather than causal effects.

## 1. Introduction

The healthcare sector faces an omnichannel imperative: patients increasingly expect seamless experiences when transitioning between online registration, face-to-face consultations, digital medical record access, and post-visit communication. ^1, 2^ In Indonesia, the National Health Insurance (Jaminan Kesehatan Nasional, JKN) ecosystem, which covers more than 260 million enrolees, ^3^ has accelerated healthcare digitalisation through the Mobile JKN application, telemedicine, and online registration systems. Although the omnichannel literature has expanded considerably in retail, ^4, 5^ banking, ^6^ and fashion, ^7^ several specific aspects of omnichannel research in healthcare remain underdeveloped. First, despite advocacy being a critical behavioural outcome in health care service ecosystems, the effect of omnichannel integration on patient advocacy has rarely been empirically tested. Second, the psychological mechanism—particularly the mediating role of brand trust—linking channel integration to advocacy has not been examined in mandatory health care contexts. Third, existing omnichannel evidence is dominated by voluntary, commercial service settings; empirical evidence from mandatory, credence-based healthcare systems, such as Indonesia’s JKN, remains scarce. ^8, 9^ This study addresses these gaps by examining the trust-mediated pathway from omnichannel integration to brand advocacy in a mandatory healthcare context, thereby advancing the literature on omnichannel services beyond its predominant focus on retail and commercial services.

This gap is not merely contextual. Health care possesses fundamental characteristics that may influence the mechanisms by which omnichannel experiences translate into patient behavioural responses. First, healthcare constitutes a credence service—its quality is difficult to evaluate even after consumption ^10^—suggesting that trust may serve as a more critical cognitive prerequisite than in search-good or experience-good contexts. Second, patients face constrained switching under the mandatory JKN system: although they can technically transfer to a different primary health care facility, their freedom of choice is limited by administrative processes and information asymmetries. Third, perceived risk in health care decisions is substantially higher than in retail purchase decisions, as it directly concerns physical well-being. ^11^ These three characteristics—credence, constraint, and risk—suggest that omnichannel integration may influence patient behaviour less through experiential value and more through trust formation, making health care a theoretically distinct setting for examining omnichannel mechanisms.

Before introducing the theoretical framework, clarifying the referent of “brand” in this study is important. In mandatory health care contexts, the notion of brand differs from that in conventional retail or commercial services because patients interact with a layered service system rather than a single commercial entity. In this study, “brand” specifically refers to the healthcare facility where patients receive services (i.e., the hospital or clinic), which functions as the proximal service provider with which patients form direct experiential and relational bonds. While BPJS Kesehatan operates as the national insurance administrator and Mobile JKN serves as a digital access platform, both function as institutional and channel infrastructure rather than the service brand itself. Therefore, brand trust and brand advocacy in this study are operationalised at the level of the health care facility as the focal brand.

Drawing on the Stimulus-Organism-Response (S-O-R) Theory, ^12^ this study positions brand trust as the organism—an internal cognitive-affective state linking the omnichannel stimulus to the advocacy response. This argument is based on commitment-trust theory, ^13^ which positions trust as a central variable in relational exchange, and service-dominant logic, ^14^ which views consumers as co-creators of value through advocacy behaviour. Rather than assuming that trust is universally superior as a behavioural driver across all service contexts, this study examines whether trust constitutes the dominant mediating mechanism in mandatory, credence-based, and high-risk healthcare—testing whether channel integration lacks a direct effect on advocacy once trust is considered.

This study contributes to omnichannel service research by identifying a trust-dominant transmission mechanism in mandatory, credence-based health care services. Specifically, it examines whether omnichannel integration is directly or primarily associated with patient advocacy through brand trust. By positioning brand trust as the organism within the S-O-R framework, the study shows that channel integration functions less as an immediate advocacy-generating experience and more as a reliability signal that must first be converted into institutional trust before advocacy emerges. These findings offer practical implications for healthcare managers by suggesting that trust-building mechanisms, such as cross-channel information consistency, transparent service processes, reliable follow-up, and responsive service recovery, should accompany investments in channel integration.

## 2. Literature review

### 2.1 Omnichannel integration in the health care sector

Omnichannel integration is defined as the synergistic management of available channels and touchpoints to optimise cross-channel customer experience and overall channel performance. ^2^ Unlike multichannel approaches, which manage channels in silos, omnichannel emphasises full integration, which eliminates barriers between channels. ^4^ In healthcare, this is manifested through the integration of registration channels (online booking, walk-in, WhatsApp), cross-platform information consistency, patient data continuity, and post-visit support through digital channels. ^1, 6, 9^ Gao et al. ^15^ found that cross-channel information consistency reduces consumer uncertainty. Beckers et al. ^16^ confirmed that integrated CCEs foster trust and engagement. The pandemic-accelerated digitalisation has further amplified the relevance of omnichannel in healthcare, as patients increasingly expect seamless transitions between digital and physical interactions. ^17^

In this study, omnichannel integration is operationalised through patient-perceived indicators that capture the healthcare-specific dimensions of channel synergy: continuity of medical and administrative information across channels, consistency of service quality between digital and physical encounters, reliability of cross-channel referral and follow-up processes, and seamlessness of transitions between Mobile JKN, facility-based services, and post-visit digital support.

This study deliberately adopts the term “omnichannel integration” rather than the more established “omnichannel retailing” Verhoef et al. ^18^ because the latter carries retail-specific connotations—product purchasing, store visits, and shopping carts—that do not translate meaningfully to healthcare service delivery. In healthcare, patients do not “retail”; they navigate a complex service ecosystem involving registration, consultation, referral, and follow-up across institutional boundaries. “Integration” foregrounds the cross-channel coordination mechanism—the construct of theoretical interest—rather than the commercial transaction context from which the concept originated. This terminological choice also aligns with the measurement approach: the instrument items ( Section 3.2) capture patient-perceived channel synergy (i.e., information consistency, data continuity, and cross-channel support) rather than purchase-related behaviours.

This conceptual framing is consistent with the growing trend of applying the omnichannel framework to various service sectors beyond traditional retail. In the Indonesian JKN context, omnichannel integration operates through a multi-layered service architecture: patients access services through the Mobile JKN application for registration and referral, BPJS Kesehatan digital platforms for insurance verification and information, telemedicine for remote consultation, WhatsApp-based facility communication, and in-person visits to primary or referral facilities. Therefore, the integration challenge in JKN is distinctive: each touchpoint is operated by a different institutional actor (the national insurer, the healthcare facility, and third-party digital service providers), yet patients perceive the service as a single continuous journey. Thus, cross-channel consistency in JKN requires synchronisation between national insurance infrastructure and facility-level service delivery, an aspect that has rarely been examined in extant omnichannel literature. Therefore, this study positions JKN as an empirical context in which omnichannel integration is shaped not only by facility-level digital capability but also by the structural features of a mandatory, multi-actor healthcare ecosystem. ^6^

### 2.2 Brand trust as an organism within the S-O-R framework

Brand trust—the willingness of consumers to rely on a brand’s ability to perform its stated function ^19^—is conceptualised in this study as the organism within the S-O-R framework: an internal cognitive-affective state that is influenced by stimulus and generates a response. ^12^ This conceptualisation draws on commitment-trust theory, ^13^ which identifies trust as a central mechanism in relational exchange. Sirdeshmukh et al. ^20^ further demonstrated that trust serves as a key driver of value and loyalty across different service contexts, supporting the generalizability of trust-based mechanisms. Trust can become a particularly salient evaluative mechanism in credence services such as health care because patients face inherent limitations in directly assessing service quality. ^10, 11^ This trust-dependent evaluation is particularly pronounced in healthcare, where patients rely on institutional signals to assess service quality. ^21, 22^ When clinical quality cannot be objectively verified by patients, they tend to rely on more observable cues—such as system orderliness, information consistency, process reliability, and cross-channel professionalism. Accordingly, trust may be viewed as an important internal mechanism for translating channel integration experiences into broader behavioural responses, including advocacy, in omnichannel health care.

### 2.3 Brand advocacy as a response

Brand advocacy—the voluntary behaviour of promoting, recommending, and defending a brand ^23, 24^—is a critical behavioural outcome in the healthcare ecosystem. Although brand advocacy conceptually overlaps with WOM, loyalty, recommendation intention, and customer retention, it remains distinct in several important ways. Whereas WOM captures any informal communication about a brand—positive, neutral, or negative—advocacy refers specifically to the brand’s active, positively balanced, and identity-laden promotion. Loyalty primarily reflects repeat-patronage behaviour or attitudinal attachment, whereas advocacy is an outward-directed behaviour towards third parties. Recommendation intention is a cognitive predisposition; advocacy is a broader behavioural construct that also encompasses brand defence despite criticism. Retention measures the continuation of the customer relationship, whereas advocacy measures the customer’s willingness to extend the brand’s reach. In mandatory health care contexts, such as JKN, this distinction is particularly important: because patients face constrained switching, retention, and loyalty metrics may be inflated by structural lock-in rather than genuine endorsement. In contrast, advocacy captures patients’ discretionary willingness to promote and defend the facility despite the absence of strong switching freedom, making it a more diagnostic indicator of authentic relational quality.

From a service-dominant logic perspective, ^14^ advocacy represents patients’ contributions as co-creators of value. Its relevance is heightened in the JKN system because facility selection decisions heavily depend on personal recommendations; the credence nature of healthcare makes others’ experiences a crucial information source; and advocacy makes an instrument of retention. ^23^

Kim and Lee ^25^ demonstrated that OCI promotes advocacy through mediation, yet the specific pathway from OCI to brand trust and then to brand advocacy in mandatory credence services has not been tested. To improve readability, this paper paraphrases the pathway as “omnichannel integration to brand trust to brand advocacy” throughout.

### 2.4 Development of hypothesis

**Figure 1.**
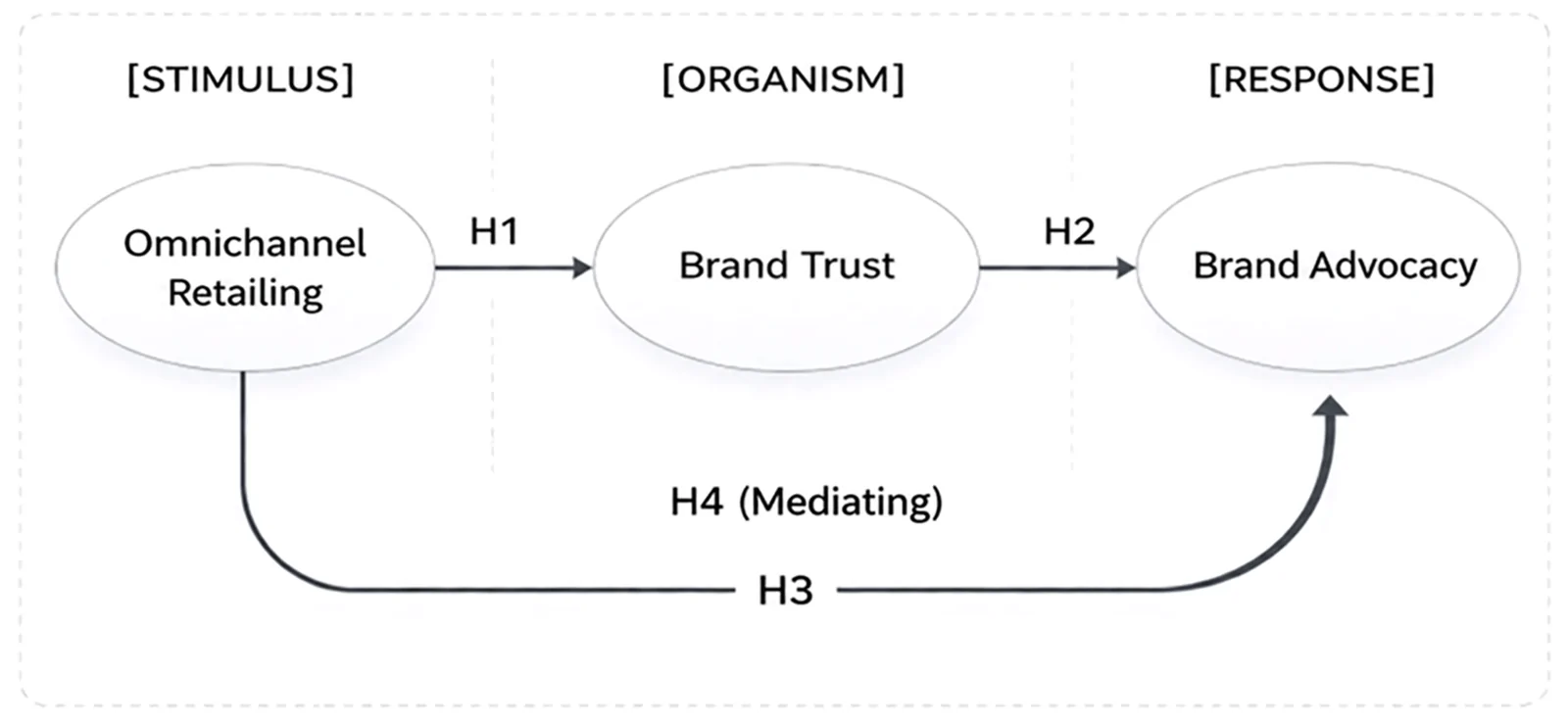
Conceptual framework: S-O-R model in omnichannel health care. ^26^

Based on the foregoing synthesis, four hypotheses are proposed, as illustrated in the conceptual framework ( Figure 1):

H1: Omnichannel integration is positively associated with brand trust. Consistent channel integration provides institutional competence and reliability signals. ^4, 15^ Cross-channel consistency serves as an important cue for trust formation in credence-service settings with high information asymmetry, as patients cannot directly evaluate clinical quality and therefore rely on peripheral cues such as system orderliness and information consistency.

H2: Brand trust is positively associated with brand advocacy. Commitment-Trust Theory predicts that relational trust promotes cooperative behaviours, including advocacy. ^13^ Empirical evidence from health care settings supports this relationship: Chang et al. ^22^ found that trust significantly predicts patient satisfaction and BI in medical encounters. This relationship is expected to be strong in mandatory health care because constrained switching makes trusting patients more likely to become active advocates rather than switch providers.

H3: Omnichannel integration is positively associated with brand advocacy. Beyond the trust pathway, omnichannel integration provides intrinsic experiential value that may directly promote advocacy—for example, through access convenience, reduced effort, and service personalisation that exceeds expectations—benefits that may independently stimulate advocacy. ^16, 25^ While brand trust is expected to explain an important indirect pathway, the direct pathway is hypothesised to coexist; the relative magnitude of these two pathways is examined empirically rather than asserted a priori.

H4: Brand trust mediates the effect of OCI on brand advocacy. Trust functions as a mediating pathway linking the omnichannel stimulus to the advocacy response within the S-O-R framework. Trust-based mediation is expected to be particularly relevant in credence-based, high-risk health care because patients face limitations in directly evaluating service quality. Accordingly, the more favourable the perceived channel integration, the greater the likelihood of trust formation, which in turn increases the willingness of patients to recommend and defend their health care provider.

## 3. Research method

### 3.1 Research design, population, and sample

This study employed a quantitative, cross-sectional survey design. Data were collected from patients at three healthcare facilities in Jakarta and Tangerang that were purposively selected to represent different levels of care and ownership types within the Indonesian JKN ecosystem: (i) UKRIDA Primaya Hospital, a private secondary/tertiary referral hospital; (ii) Kalideres General Hospital, a public secondary referral hospital; and (iii) Klinik Sehatku, an independent primary care clinic located in Pasar Kemis, Tangerang Regency. This three-facility design captures variation across (a) levels of care (primary vs. secondary/tertiary), (b) ownership types (private, public, and independent), and (c) patient segments served (JKN-insured and general). The omnichannel service environment at each facility was documented through researcher observation and a structured availability check of patient-facing channels at each site—covering Mobile JKN integration, facility website, WhatsApp service, online appointment booking, and walk-in service—conducted prior to data collection. This check confirmed that all of these core patient-facing channels were available at all three facilities. Patient-facing access to electronic medical records, by contrast, was only partial at all three sites: patients could view their visit history through the Mobile JKN application, but complete electronic medical records were not directly accessible to patients. Cross-facility variation therefore lay primarily in levels of care, ownership type, and depth of channel implementation rather than in the availability of the channels themselves. Responses were distributed approximately evenly across the three facilities (UKRIDA Primaya Hospital: n = 77; Kalideres General Hospital: n = 77; Klinik Sehatku: n = 76) to avoid over-representation of any single facility. Data collection procedures (sampling approach, inclusion screening, questionnaire administration mode, and supervising research assistants’ protocol) were standardised across all three facilities to ensure cross-site comparability.

Consistent with the cross-sectional design, each respondent completed the questionnaire at a single point in time: questionnaires were distributed to patients immediately after they had completed an omnichannel service journey (i.e., online registration via Mobile JKN, website, or WhatsApp followed by an in-person visit) and after they had consulted with the physician. Patients were approached by nurses immediately after this consultation: the nurse briefly explained the study and asked whether the patient was willing to complete the questionnaire, and questionnaires were administered only to patients who agreed. Administering the questionnaire at this point ensured that respondents could reflect on a complete, recently-experienced omnichannel encounter rather than hypothetical or distant past interactions.

Purposive sampling was applied with the following inclusion criteria: (1) aged ≥18 years; (2) having used services at the facility at least twice within the past 12 months—to ensure that respondents had sufficient repeated, cross-channel exposure to meaningfully evaluate channel integration, information consistency, and continuity of experience over time; and (3) having accessed services through digital channels (mobile application, website, or WhatsApp) in addition to physical visits, to ensure first-hand omnichannel exposure relevant to the focal construct.

The main data collection was conducted from March 11, 2026, to March 30, 2026, immediately following the pre-test phase (n = 50), which was conducted from March 8–10, 2026 and is reported in Section 3.7. A total of 230 questionnaires were collected; all were complete and usable, none were excluded prior to analysis, and the sample exceeds the minimum threshold of 110 respondents based on PLS-SEM power analysis. This 100% usable-questionnaire rate reflects the supervised, in-person administration mode, in which research assistants checked questionnaires for completeness at the point of return. However, because the number of patients who declined the nurses’ invitation was not recorded, a conventional response rate (completed questionnaires relative to patients approached) cannot be computed, and this figure should not be read as indicating that no patients declined to participate.

Ethical considerations were observed throughout: the study received ethical approval from the Research Ethics Committee of Universitas Esa Unggul (Komisi Etik Penelitian Universitas Esa Unggul; Approval No. 0925–02.059/DPKE-KEP/FINAL-EA/UEU/II/2026, dated 20 February 2026) prior to fieldwork. Participation was voluntary, and written informed consent was obtained from all respondents. Respondents were informed of their right to withdraw at any time without consequence. Anonymity was preserved by removing personal identifiers from the dataset, and responses were kept confidential through restricted-access data storage. The de-identified survey dataset underlying the results reported in this article is openly available in figshare. ^38^ No incentives were provided that could compromise voluntariness. ^27^

### 3.2 Instrument development

The instrument comprised 17 items, which were measured using a 5-point Likert scale (1 = strongly disagree; 5 = strongly agree). The omnichannel integration (10 items, OCI1–OCI10) was adapted from Verhoef et al. ^2^ and Shi et al., ^1^ covering channel integration, information consistency, data continuity, and cross-channel support. These items capture the perceived quality of integration across channels rather than the mere availability of individual digital services: the indicators reference whether information is consistent across channels, whether medical and administrative records remain continuous when patients move between channels, and whether patients feel supported when switching channels—dimensions that presuppose, but go beyond, simple multichannel access. Channel availability itself was documented separately at the facility level ( Section 3.1). Brand trust (4 items, BT1–BT4) was adapted from Holbrook et al., ^19^ trust, reliability, honesty, and safety. Brand advocacy (3 items, BA1–BA3) was adapted from Kemp et al. ^23^ and Stokburger-Sauer et al. ^24^: willingness to try new programmes and services, positive word-of-mouth, and willingness to give the provider another chance. The third item (BA3) was retained because the advocacy literature treats defence of and continued commitment to a provider as advocacy behaviour, and its wording is revisited in the Limitation section.

Illustrative items include the following: Omnichannel integration: “The information I receive about services at this facility is consistent across channels (e.g., Mobile JKN, website, WhatsApp, and in-person)” (OCI-information consistency) and “My medical and administrative records are continuous and accessible regardless of the channel I use” (OCI-data continuity). Brand trust: “I trust this health care facility to deliver services reliably and safely” (BT-reliability/safety). Brand advocacy: “I want to try new health programmes and services introduced by the facility” (BA-engagement) and “I am willing to give the facility another chance even if it has disappointed me” (BA-forgiveness). The full list of items is presented alongside the descriptive statistics in Section 4 to enable interpretation of the construct measurement.

### 3.3 Forward translation

The instrument adaptation followed forward translation procedures. ^28^ Two independent translators proficient in both languages and familiar with management and health care terminology translated English-language items into Bahasa Indonesia. The consolidated translation maintained semantic and conceptual equivalence, with contextual adaptations from retail to health care (e.g., “purchase” became “service visit”; “store” became “health care facility”).

### 3.4 Back translation

The Bahasa Indonesia translation was back-translated into English by a third independent translator that was blind to the original instrument to verify meaning equivalence. ^28^ Then, the back-translated version was compared with the original English version. The comparison revealed substantial equivalence across all 17 items, with no significant discrepancies in meaning. Minor wording adjustments were made to improve clarity without altering the underlying constructs.

### 3.5 Validity

The questionnaire was administered to five patients who met the inclusion criteria to evaluate readability, language clarity, and contextual relevance. Based on their feedback, editorial adjustments were made to omnichannel integration items requiring technical terminology simplification without altering measurement substance.

### 3.6 Validity of content

Expert judgement was conducted by a research methodology and marketing scholar. Each item was evaluated on the following: (a) relevance to construct definition; (b) clarity of formulation; and (c) appropriateness for the Indonesian health care context. Revisions included converting negatively worded items to positive and replacing retail terminology with health care terminology. All 17 items were deemed valid.

### 3.7 Pre-test (n = 50)

A pre-test was conducted with 50 respondents from 8–10 March 2026, separate from the main sample, primarily to evaluate instrument readability, item clarity, and initial indicator adequacy before main data collection. Preliminary evaluation showed that all the items met the minimum thresholds for outer loading, reliability, and AVE. Pre-test results were treated as evidence of initial adequacy and instrument refinement and not as the primary basis for substantive conclusions. Accordingly, the main validity and reliability evaluation in this study relies on a larger and more heterogeneous sample (n = 230).

### 3.8 Common method bias (CMB)

CMB was addressed through both procedural and statistical approaches. Procedurally: (a) The questionnaire’s introduction stated that there were no right or wrong answers; (b) the item wording was varied to reduce automatic response patterns; (c) independent, mediator, and dependent variable sections were visually separated; and (d) scale anchor points were kept consistent to reduce acquiescence bias. ^29^

Statistically, four complementary procedures were applied to evaluate the potential CMB. First, Harman’s single-factor test showed that a single factor explained 48.12% of the total variance, which is below the conventional 50% threshold. As highlighted by Fuller et al. ^30^ and Podsakoff et al., ^29^ this test has low sensitivity and specificity and should not be treated as conclusive evidence of bias. Second, the correlation matrix procedure ^31^ showed no inter-construct correlation exceeding 0.90, with the highest correlation between BT and BA (r = 0.684). Third, the inner VIF for the structural model was 2.047, which is well below the threshold of 5.0. ^27^ Fourth, the model was re-estimated using an unmeasured latent method factor. ^29^ All indicators were allowed to load on both their theoretical constructs and a common method factor. The common method factor accounted for 5.8% of the total variance, and the structural path coefficients remained significant, changing by less than 0.02; R ^2^ values changed by 0.01. Taken together, the procedural remedies, Harman’s single-factor test, inter-construct correlation assessment, inner VIF evaluation, and unmeasured latent method factor analysis suggest that the observed relationships are unlikely to be fully accounted for by common method bias.

### 3.9 Analytical technique

PLS-SEM with 5,000 bootstrap subsamples was employed for analysis. The evaluation of the outer model included convergent validity (outer loading >0.70; AVE > 0.50), discriminant validity (Fornell-Larcker criterion; cross-loadings; HTMT inference with bootstrapped CI), and reliability (Cronbach’s α > 0.70; CR > 0.70). The inner model evaluation included the path coefficients, R ^2^, f ^2^, Stone-Geisser’s Q ^2^ obtained through blindfolding with an omission distance of 7, and mediation testing using the specific indirect effect, VAF, and bias-corrected confidence intervals. An alternative 2-factor model (BT + BA merged) was compared against the 3-factor model as a robustness check. Control variables (age, gender, and education) were included to test the stability of the core relationships. Age was controlled because digital channel familiarity and adoption patterns vary across generational cohorts, ^32^ which may confound the perception of omnichannel integration. Education was controlled because health literacy, digital literacy, and the ability to evaluate cross-channel information consistency tend to vary with educational attainment and may also influence the cognitive processing underlying trust formation. Gender was controlled because prior healthcare-service research has reported gender-related differences in healthcare utilisation patterns, communication preferences, and trust formation in patient–provider relationships. ^33^ Therefore, including gender as a control guards against spurious attribution of these baseline differences to the focal omnichannel–trust–advocacy relationships.

## 4. Findings

### 4.1 The Respondent profile

Table 1 presents the profiles of the 230 respondents. The age distribution was relatively balanced: 18–30 years (31.3%), 31–45 years (27.4%), 46–60 years (21.7%), and above 60 years (19.6%). Gender composition was balanced (49.6% male and 50.4% female). Educational profiles represented all strata: high school (24.8%), diploma (25.2%), bachelor’s degree (28.7%), and postgraduate (21.3%). Within this digitally active sample (all 230 respondents had used at least one digital channel per the inclusion criterion), the channels used were Mobile JKN, WhatsApp registration, and the facility website. No respondent relied exclusively on physical registration, confirming the multi-channel nature of patient interactions in the context of this study.

**Table 1.** Respondent Profile (n = 230).

Characteristic	Category	n	%
Age	18–30 years	72	31.3
	31–45 years	63	27.4
	46–60 years	50	21.7
	>60 years	45	19.6
Gender	Male	114	49.6
	Female	116	50.4
Education	High School	57	24.8
	Diploma	58	25.2
	Bachelor	66	28.7
	Postgraduate	49	21.3

Table 2 presents the descriptive statistics for all 17 questionnaire items across three constructs: OCI, brand trust, and brand advocacy. For OCI (OCI, 10 items), the construct-level mean was 3.776 on a 5-point Likert scale, indicating a moderately positive perception of channel integration among respondents. The highest-rated item was OCI8 (“Patients can access integrated history of their healthcare utilisation”; M = 3.913, SD = 0.926), with 70.9% of respondents expressing agreement or strong agreement. The lowest-rated item was OCI5 (“Facility allows patients to self-check-in for appointments made through the online system”; M = 3.683, SD = 1.069), where only 56.5% agreed or strongly agreed, suggesting that self-check-in functionality represents an area for improvement in the facilities under study. Across all OCI items, the proportion of respondents expressing disagreement (Strongly Disagree + Disagree) ranged from 7.8% to 13.9%, while 56.5% to 70.9% expressed agreement or strong agreement.

**Table 2.** Descriptive Statistics of Questionnaire Items (n = 230).

	Description	Mean	Standard Deviation	Strongly Disagree (1)	Disagree (2)	Neutral (3)	Agree (4)	Strongly Agree (5)
OCI1	Health service promotions displayed on the website/app	3.826	0.996	4	20	53	88	65
OCI2	Address and contact information on the website/app	3.843	0.994	4	18	56	84	68
OCI3	Available services listed on the website/app	3.761	1.011	5	21	58	86	60
OCI4	Services after completing the online registration	3.748	1.052	7	22	55	84	62
OCI5	Self-check-in for online appointments	3.683	1.069	5	27	68	66	64
OCI6	Selecting service units during online registration	3.783	1.026	3	25	58	77	67
OCI7	Integrated Visit History across Channels	3.700	1.003	5	24	58	91	52
OCI8	Access to integrated health care utilisation history	3.913	0.926	2	16	49	96	67
OCI9	Complaint handling for online registered services	3.726	1.097	7	25	61	68	69
OCI10	Accessible online and in-person post-service support	3.774	0.985	4	19	62	85	60
BT1	I trust this health care facility.	3.822	1.006	5	19	53	88	65
BT2	I rely on this health care facility	3.774	1.062	11	12	59	84	64
BT3	This is an honest facility	3.804	1.062	9	15	57	80	69
BT4	This is a safe facility.	3.887	1.017	7	14	49	88	72
BA1	Try new programmes/services introduced by the facility	3.809	1.148	15	12	51	76	76
BA2	Speak positively to friends and family	3.817	1.106	7	26	45	76	76
BA3	Give facility another chance despite disappointment	3.770	1.038	4	24	60	75	67

For brand trust (BT, 4 items), the construct-level mean was 3.822, which was slightly higher than that of OCI. BT4 (“This healthcare facility is a safe facility”; M = 3.887, SD = 1.017) received the highest endorsement, with 69.6% of respondents agreeing or strongly agreeing, indicating that perceived safety is the strongest dimension of facility trust. BT2 (“I rely on this healthcare facility”; M = 3.774, SD = 1.062) scored lowest within the trust construct, suggesting that reliance—implying habitual dependence—is somewhat weaker while patients generally trust the facility. The standard deviations across BT items (1.006–1.062) were relatively uniform, indicating comparable response variability.

The construct-level mean for brand advocacy (BA, 3 items) was 3.799. BA2 (“I speak positively about the healthcare facility to friends and family”; M = 3.817, SD = 1.106) and BA1 (“I want to try new health programmes and services introduced by the facility”; M = 3.809, SD = 1.148) received nearly identical endorsement levels (66.1% agreement/strong agreement each). BA3 (“I am willing to give the facility another chance even if it has disappointed me”; M = 3.770, SD = 1.038) scored lowest at 61.7% agreement, which is notable because this item captures the willingness to give the provider another chance after disappointment—a behaviourally demanding, forgiveness-oriented form of advocacy that goes beyond simple recommendation. The relatively high standard deviation of BA1 (1.148) indicates greater response dispersion, reflecting heterogeneous willingness to explore new facility programmes.

Overall, all 17 items exhibited means above the midpoint of the scale (3.00), confirming that the respondents held moderately positive perceptions across the three constructs. The construct-level ordering—brand trust (M = 3.822) > brand advocacy (M = 3.799) > omnichannel integration (M = 3.776)—suggests that patients’ affective evaluations (trust) slightly exceeded their perceptions of channel integration and advocacy behaviours, a pattern consistent with the hypothesised mediating role of trust in the omnichannel integration to brand trust to brand advocacy pathway.

### 4.2 Outer model evaluation

All 17 indicators exhibited outer loadings above the 0.70 threshold (range: 0.704–0.877; Table 3). AVE for all constructs exceeded 0.50: OCI (0.548), BT (0.687), and BA (0.752). Cronbach’s alpha (0.835–0.908) and composite reliability (0.898–0.924) exceeded 0.70. Convergent validity and internal consistency reliability were confirmed.

**Table 3.** Convergent validity and reliability (n = 230).

Construct	Item	Loading	α	CR	AVE	Status
OCI (X)	OCI1	0.761	0.908	0.924	0.548	Valid
	OCI2	0.741				
	OCI3	0.716				
	OCI4	0.704				
	OCI5	0.722				
	OCI6	0.794				
	OCI7	0.733				
	OCI8	0.732				
	OCI9	0.768				
	OCI10	0.729				
BT (M)	BT1	0.821	0.848	0.898	0.687	Valid
	BT2	0.845				
	BT3	0.827				
	BT4	0.823				
BA (Y)	BA1	0.870	0.835	0.901	0.752	Valid
	BA2	0.877				
	BA3	0.855				

Criteria: Loading >0.70; α > 0.70; CR > 0.70; AVE > 0.50 (Hair et al., 2019)

### 4.3 Discriminant validity

The discriminant validity was assessed using three complementary approaches. First, cross-loading analysis ( Table 4) showed that all 17 indicators loaded highest on their own latent construct, with no cross-loading exceeding own-loading on any other construct—satisfying the cross-loading validity criterion.

**Table 4.** Cross-Loadings Matrix (n = 230).

Item	OCI	BT	BA
OCI1	**0.761**	0.553	0.342
OCI2	**0.741**	0.564	0.372
OCI3	**0.716**	0.575	0.431
OCI4	**0.704**	0.469	0.383
OCI5	**0.722**	0.506	0.322
OCI6	**0.794**	0.558	0.361
OCI7	**0.733**	0.519	0.428
OCI8	**0.732**	0.477	0.365
OCI9	**0.768**	0.558	0.375
OCI10	**0.729**	0.511	0.309
BT1	0.608	**0.821**	0.556
BT2	0.568	**0.845**	0.598
BT3	0.621	**0.827**	0.533
BT4	0.574	**0.823**	0.581
BA1	0.435	0.600	**0.870**
BA2	0.404	0.569	**0.877**
BA3	0.458	0.611	**0.855**

Bold = loading on the construct. All items load the highest on their own construct.

Second, the Fornell-Larcker criterion ( Table 5) indicated that the square root of AVE (√AVE) for each construct was generally larger than the corresponding inter-construct correlations. The BT–BA pair exhibited a moderate-to-high correlation (r = 0.684), although √AVE for BA (0.867) remained larger than this correlation, satisfying the Fornell-Larcker criterion. Conceptually, these two constructs are substantively distinct: brand trust is an evaluative relational belief—an evaluative conviction regarding a healthcare facility’s reliability and integrity—whereas brand advocacy is a voluntary promotive behaviour—the volitional act of recommending and defending. This conceptual distinction is important because the mediation claim depends on the separability of the mediator and the outcome.

**Table 5.** Fornell-Larcker Criterion.

Construct	OCI	BT	BA
OCI	**0.740**		
BT	0.715	**0.829**	
BA	0.498	0.684	**0.867**

Diagonal (bold) = √AVE; off-diagonal = inter-construct correlations

Third, HTMT inference with 5,000 bootstrap subsamples ( Table 6) showed: HTMT (OCI–BT) = 0.814 [95% CI: 0.745, 0.873] and HTMT (OCI–BA) = 0.571 [95% CI: 0.458, 0.679]—both below the 0.90 threshold and with CIs not including 1.0. The BT–BA pair yielded HTMT = 0.812 (95% CI: 0.735, 0.879), which was also below 0.90 (95% CI not including 1.0). All construct pairs met the HTMT-based discriminant validity criterion.

**Table 6.** HTMT Inference (Bootstrapped 95% CI).

Pair	HTMT	95% CI Lo	95% CI Hi
OCI ↔ BT	0.814	0.745	0.873
OCI ↔ BA	0.571	0.458	0.679
BT ↔ BA	0.812	0.735	0.879

Threshold: HTMT < 0.90; CI does not include 1.0 ^36^

A model comparison was conducted to further clarify BT-BA discriminant status. The 3-factor model (OCI, BT, and BA separately) was compared with a 2-factor model (BT and BA merged). The 3-factor model produced lower residual sum of squares and higher AVE for both BT (0.687) and BA (0.752) than the merged BT_BA construct (0.604). The superiority of the 3-factor model, combined with clean cross-loadings, supports the retention of two separate constructs. The discriminant validity between BT and BA was confirmed through all three approaches (cross-loadings, Fornell-Larcker, and HTMT), thereby supporting the retention of the 3-factor model.

Additionally, an AVE–SV analysis was conducted to further assess the discriminant validity. As shown in the AVE–SV table (relocated here from Section 4.6 for logical grouping with the other discriminant validity diagnostics), the AVE for each construct exceeded the highest shared variance with any other construct, reinforcing the conclusion that the constructs are empirically distinct.

### 4.4 Inner model evaluation and hypothesis testing

Table 7 summarises the results of the hypothesis testing. H1 was supported (β = 0.715; t = 21.284; 95% CI [0.644, 0.776]). Omnichannel integration was positively and significantly associated with brand trust, with a large effect size (f ^2^ = 1.047).

**Table 7.** Hypothesis testing results (5,000 bootstrap subsamples).

H	Path	β	SE	t-stat	95% CI	Result
H1	OCI to BT	0.715	0.034	21.284 ***	[0.644,0.776]	Supported
H2	BT to BA	0.671	0.065	10.358 ***	[0.539,0.792]	Supported
H3	OCI to BA	0.019	0.071	0.263 ns	[−0.119,0.157]	Not Supp.
H4	OCI to BT to BA	0.480	0.055	8.752 ***	[0.372,0.586]	Supported

*** p < 0.001; ns = not significant; CI = bias-corrected confidence interval; H4 = -specific indirect effect

H2 was supported (β = 0.671; t = 10.358; 95% CI [0.539, 0.792]). Brand trust was significantly positively associated with brand advocacy, with a large effect size (f ^2^ = 0.413).

H3 was not supported (β = 0.019; t = 0.263; p = 0.793; 95% CI [−0.119, 0.157]). The direct effect of OCI on brand advocacy was not statistically significant when brand trust was included in the model, with a negligible effect size (f ^2^ = 0.000).

As shown in Table 8, R ^2^ for brand trust and brand advocacy was 0.511 and 0.468, respectively, indicating moderate-to-substantial explanatory power. Adjusted R ^2^ values were 0.509 and 0.464. The effect size of OCI-to-BT was large (f ^2^ = 1.047), BT-to-BA was large (f ^2^ = 0.413), and OCI-to-BA was negligible (f ^2^ = 0.000). This pattern is consistent with trust serving as a meaningful mediator in the model. Stone-Geisser Q ^2^ values obtained via blindfolding (omission distance D = 7) were 0.350 for brand trust and 0.349 for brand advocacy, both exceeding the zero threshold and indicating medium predictive relevance. ^27^

**Table 8.** Structural model evaluation.

Endogenous	R ^2^	Adj. R ^2^	f ^2^	Q ^2^
Brand Trust	0.511	0.509	1.047	0.350
Brand Advocacy	0.468	0.464	0.413/0.000	0.349

f ^2^: large > 0.35; medium > 0.15; small > 0.02. ^37^ f ^2^ BA: BT-to-BA/OCI-to-BA

### 4.5 Mediation analysis

The indirect effect was significant (β = 0.480; t = 8.752; 95% CI [0.372, 0.586]). VAF of 96.2% indicates a full-mediation pattern in the tested model (Hair et al., ^27^ classify VAF ≥ 80% as full mediation): The association between OCI and advocacy operates predominantly through trust. This finding supports the S-O-R framework in omnichannel healthcare, suggesting that trust functions as the primary mediating pathway in the tested model.

### 4.6 Robustness check: Control variables

The model was re-estimated using age, gender, and education as the control variables. The OCI-to-BT and BT-to-BA ratios remained statistically significant (p < 0.001), whereas the OCI-to-BA ratio remained non-significant (p > 0.05). None of the control variables were significantly associated with brand trust or brand advocacy (p > 0.05). R ^2^ values changed by less than 0.01 after including the controls, indicating that the demographic factors did not materially alter the explanatory power or the pattern of relationships.

## 5. Discussion

### 5.1 Omnichannel integration and brand trust (H1)

The strong positive association between OCI and brand trust (β = 0.715; f ^2^ = 1.047) provides compelling evidence that channel integration functions as a trust signal in mandatory credence services. This finding is consistent with the argument that in credence services—where patients cannot directly evaluate clinical quality—they rely on peripheral cues such as system orderliness, information consistency, and cross-channel professionalism to assess institutional trustworthiness. The result aligns with the findings of Juaneda-Ayensa et al., ^5^ who showed that channel integration enhances cognitive and affective brand evaluations, and with Weippert, ^34^ who demonstrated that online–offline integration shapes brand-level outcomes through perceived service quality. Our contribution extends this evidence to the mandatory credence-service domain, where trust formation has previously been theorised as institution- and relationship-driven ^21, 22^ rather than channel-driven.

The magnitude of the OCI-to-BT path (β = 0.715) is notably larger than the effects typically reported in retail omnichannel studies, ^1, 4^ suggesting that channel-integration cues may carry disproportionate diagnostic weight in high-risk credence settings. This may reflect the multi-actor service architecture in the JKN context: patients interact with national insurance platforms (Mobile JKN, BPJS Kesehatan), facility-level digital services, and in-person clinical encounters. When these disparate touchpoints exhibit consistency—for instance, when appointment information on Mobile JKN matches the facility reception process—patients may interpret this as a signal that the healthcare ecosystem is well-coordinated and therefore trustworthy. This interpretation is supported by the item with the highest mean score (OCI8: access to integrated healthcare utilisation history; M = 3.913), as data continuity across channels is precisely the type of system-level cue that signals institutional reliability. Nevertheless, the cross-sectional design and single-source measurement warrant cautious interpretation of the magnitude of the effect.

### 5.2 Brand trust and brand advocacy (H2)

The significant trust-to-advocacy path (β = 0.671; f ^2^ = 0.413) extends the relevance of commitment-trust theory (Morgan & Hunt, 1994) to the mandatory health care domain. This finding is consistent with those of Sirdeshmukh et al. ^20^ and Kemp et al., ^23^ who established trust as a robust antecedent of advocacy and BSP in voluntary-choice service settings. Our contribution is to show that this trust-to-advocacy link persists in a mandatory healthcare context—a finding that is theoretically non-trivial because mandatory enrolment could plausibly attenuate the diagnosticity of advocacy as a behavioural outcome. Patients still engage in advocacy behaviours (positive word-of-mouth, willingness to try new programmes and services, and willingness to give the provider another chance) despite limited switching freedom, suggesting that advocacy in this context reflects genuine relational evaluation rather than captive loyalty.

This result is particularly meaningful when interpreted alongside the descriptive statistics. The advocacy item with the lowest agreement rate was BA3 (“I am willing to give the facility another chance even if it has disappointed me”; M = 3.770, 61.7% agreement)—an item that captures the willingness to forgive service failures, the most behaviourally demanding form of advocacy. The fact that even this demanding dimension scored above the scale midpoint and that trust explains a substantial proportion of its variance suggests that trust in mandatory health care serves as a buffer against service failures. This departs from the concern raised in Section 2.3 that structural lock-in may inflate retention metrics in mandatory schemes; advocacy, being discretionary and outward-directed, is less susceptible to such inflation.

### 5.3 Null direct effect and full mediation (H3 and H4)

The non-significant direct effect of omnichannel integration on brand advocacy (β = 0.019; p = 0.793; f ^2^ = 0.000), combined with the significant indirect effect through brand trust (β = 0.480; VAF = 96.2%), constitutes the central finding of this study. The pattern is consistent with full mediation: within the tested model, the association between OCI and advocacy is almost entirely accounted for by trust. This contrasts with Kim and Lee, ^25^ who reported a significant direct effect of omnichannel experience on advocacy in the retail context and suggested that the omnichannel-to-advocacy pathway is context-dependent—operating differently in credence-based mandatory services compared to voluntary experience-good contexts.

This finding is theoretically consistent with the credence-service argument articulated in Sections 1 and 2: the experiential value of omnichannel integration alone is insufficient to generate advocacy in healthcare settings where patients cannot directly evaluate clinical quality (Darby & Karni, 1973). ^10^ Instead, omnichannel cues must first be cognitively processed into trust before they can be translated into behavioural responses. This result is consistent with Akter et al., ^35^ who argued that quality cues in transformative health care service systems require cognitive intermediation before generating behavioural outcomes. Empirically, our finding provides the first direct evidence that the full-mediation form of the S-O-R framework is most appropriate for omnichannel integration in mandatory credence services—a methodological refinement over the partial-mediation specifications that have dominated retail omnichannel research. At the same time, this interpretation is bounded by the tested model: trust was the only mediator specified, and alternative mechanisms—such as satisfaction, perceived service quality, perceived control, convenience, or perceived risk reduction—were not included and may also transmit part of the omnichannel–advocacy association. The full-mediation pattern should therefore be read as evidence of trust’s centrality within this specification rather than as proof that omnichannel integration operates exclusively through trust.

This full-mediation finding suggests that investing in digital channel features (e.g., Mobile JKN integration, WhatsApp-based communication, and online appointment booking) may not directly stimulate patients to recommend or defend their healthcare facility. Rather, the mechanism operates indirectly: channel integration builds trust by signalling institutional coordination and reliability, and patients subsequently translate this trust into advocacy behaviours. This has a clear strategic implication: omnichannel investment without concurrent trust-building efforts—such as transparent communication, service recovery protocols, and consistent cross-channel information—is unlikely to generate patient advocacy.

### 5.4 Theoretical and managerial implications

Theoretically, this study advances omnichannel service theory by showing that in constrained credence-service settings, channel integration does not appear to operate as an advocacy-generating experience by itself; rather, it functions as a reliability signal that is cognitively converted into institutional trust before advocacy emerges. First, the study identifies a trust-dominant transmission mechanism in which brand trust serves as the primary pathway linking omnichannel integration to brand advocacy by demonstrating a significant indirect effect and a non-significant direct effect. Second, the findings refine the S-O-R framework in a healthcare context by demonstrating that the organism component—brand trust—plays a central explanatory role in translating channel-related stimuli into advocacy responses. Third, the study extends omnichannel theorising beyond voluntary-choice retail and hospitality settings by situating these dynamics within mandatory healthcare, where provider choice is structurally constrained. Because this study does not directly compare service contexts, these contributions should be interpreted as mechanism identification within a mandatory health care context rather than definitive evidence of contextual differences.

From a managerial perspective, channel integration investment is associated with operational efficiency and patient trust formation. Cross-channel information consistency, patient data continuity, ease of transition between digital and physical channels, and communication responsiveness serve as important signals shaping perceptions of institutional trustworthiness in services where patients cannot directly evaluate quality. In practical terms, this encompasses the synchronisation of online-offline appointment scheduling systems, post-visit communication through digital channels, and the integration of digital medical records accessible to patients across platforms. Additionally, the findings suggest that TB should be positioned as a strategic pillar for promoting patient advocacy. Therefore, health care facility managers may therefore consider structured patient advocacy programmes—such as referral systems, digital testimonials, and patient community development—as means to translate trust into more active recommendation behaviour. The trust-dominant mechanism further underscores that omnichannel investment without trust-building efforts is unlikely to directly generate patient advocacy.

## 6. Conclusion

This study supports the applicability of the S-O-R framework in OCH. Three hypotheses (H1, H2, and H4) were supported, whereas H3 (the direct effect) was not. The main finding is that brand trust emerges as the central mediating mechanism: once trust is considered, the direct effect of omnichannel integration on advocacy becomes statistically negligible. The model explains 46.8% of variance in brand advocacy. This result is consistent with the argument that in credence-based, high-risk healthcare, cognitive evaluation in the form of trust is the dominant pathway through which omnichannel integration is associated with patient advocacy. However, inferences regarding whether this pattern differs from non-credence contexts still require direct comparative testing.

## 7. Limitation and further research

Several limitations should be acknowledged. First, the cross-sectional design limits causal inference; all reported relationships should therefore be read as associations. This caution applies especially to the mediation analysis: mediation tested with cross-sectional data cannot demonstrate temporal ordering, so the full-mediation pattern is consistent with—rather than demonstrative of—the hypothesised omnichannel integration → trust → advocacy sequence. Longitudinal or experimental designs are needed to establish temporal ordering. Second, even though our CMB diagnostics (Harman’s test, correlation matrix procedure, inner VIF, and unmeasured latent method factor analysis) did not reveal serious threats, the single-source, single-timepoint design means that some coefficient inflation due to common source variance cannot be completely ruled out. Therefore, we interpret our coefficients as relative indicators of association rather than precise estimates free from method bias.

Third, although the HTMT between brand trust and brand advocacy (0.812; 95% CI: 0.735–0.879) meets the conservative 0.90 threshold and the square roots of AVE exceed inter-construct correlations ( Table 9), the BT–BA correlation (r = 0.684) and moderate cross-loadings (e.g., BA1 loads 0.600 on BT) indicate conceptual proximity. We re-estimated a 2-factor model combining BT and BA. The 3-factor model produced higher AVE for both BT (0.687) and BA (0.752) than the merged BT_BA construct (0.604), and the model comparison reported in Section 4.3 (lower residual sum of squares; clean cross-loadings) favoured the retention of the two separate constructs. These PLS-SEM-appropriate diagnostics, rather than covariance-based Δχ ^2^ difference testing, support discriminant validity. However, to sharpen the distinction, future research should refine item wording. For instance, the item ‘I am willing to give another chance to my health care provider” (BA3) may capture forgiveness rather than advocacy and could be revised or omitted in future studies.

**Table 9.** AVE–SV Analysis.

Construct	AVE	Highest SV	DV?
OCI	0.548	0.511	Yes
BT	0.687	0.468	Yes
BA	0.752	0.468	Yes

DV confirmed: AVE > highest shared variance for all constructs

Fourth, the study sample is a pooled sample from three health care facilities with different levels of digitalisation, ownership types, and patient profiles. The reported findings represent an aggregate cross-institutional pattern, not a mechanism that is necessarily uniform across every institution, because facility identity was not modelled as institutional controls, dummy variables, or analysed through multi-group comparison. Inter-facility heterogeneity may affect relationship estimates. Fifth, self-selection in digital channel usage may introduce endogeneity concerns that have not been fully addressed. Relatedly, because refusals at the recruitment stage were not recorded, a conventional response rate cannot be reported, and the sample may over-represent patients who are more digitally literate or more favourably disposed towards their facility; the reported associations should be read with this potential selection bias in mind.

Future research directions include: (a) testing moderators such as perceived risk, digital literacy, and service type (JKN-insured vs. general) to identify when trust’s role becomes more pronounced; (b) longitudinal or experimental designs; (c) multi-site studies with institutional controls, fixed effects, or multi-group analysis to capture inter-facility heterogeneity; (d) integration of additional constructs from the broader model such as SERVQUAL, consumer brand engagement, and brand relationship quality; and (e) direct comparison between credence services and experience services to test whether trust centrality is indeed higher in credence contexts.

## Data Availability

Figshare: Patient Survey Omnichannel Healthcare Brand Trust Support Dataset. https://doi.org/10.6084/m9.figshare.32356992.v2. ^38^ This project contains the de-identified patient-survey responses (n = 230) for the 17 questionnaire items measuring omnichannel integration, brand trust, and brand advocacy that underlie all analyses reported in this article, including the descriptive statistics in Table 2 and the PLS-SEM results in Section 4. The dataset is accompanied by a codebook documenting each variable: variable names, source columns, full item wording, response coding (five-point Likert scale), and the demographic and screening variables. Data are available under the terms of the Creative Commons Attribution 4.0 International license (CC-BY 4.0). Figshare: Survey Instrument and Informed Consent Form. https://doi.org/10.6084/m9.figshare.32526855. ^39^ This project contains the full survey instrument used in this study, comprising the 17 questionnaire items measuring omnichannel integration, brand trust, and brand advocacy, together with the participant information sheet and informed-consent form administered to respondents (in Indonesian, the original language, with English translation). Data are available under the terms of the Creative Commons Attribution 4.0 International license (CC-BY 4.0).
