# Supplementary material for: Trust as an Omnichannel-to-Advocacy Transmission Mechanism in Mandatory Health care Services: Empirical Evidence from Indonesia
Source: F1000Res. 2026 Aug 6;15:946. Originally published 2026 Jun 16. [Version 3] doi: 10.12688/f1000research.183317.3 (PMC13560807; doi:10.12688/f1000research.183317.3)
Supplement: Peer review history

# Reviewer report: Reviewer response for version 3

**DOI:** 10.5256/f1000research.207392.r502680

**Published:** 2026-09-08

**Authors:** Adilla Anggraeni

**Affiliations:** 1 Bina Nusantara University, Jakarta, Indonesia

The report offers interesting insights on the application of omnichannel service theory in the context of health services. It is advised that the authors discuss how the services may differ compared to the other type of services. For example, there is an element of co-creation which necessitates the element of trust in the relationship. 

 It is also important to note that healthcare services' service failures may be severe and hence it is understandable that service recovery may not be deemed sufficient to remedy the service failures. 

 The constrained credence-service settings also offer insights into how healthcare services could provide information to the patients beforehand, especially related to the service providers, healthcare professionals' expertise, and possibility of testimonials.

Is the work clearly and accurately presented and does it cite the current literature?

Yes

If applicable, is the statistical analysis and its interpretation appropriate?

Yes

Are all the source data underlying the results available to ensure full reproducibility?

Yes

Is the study design appropriate and is the work technically sound?

Yes

Are the conclusions drawn adequately supported by the results?

Yes

Are sufficient details of methods and analysis provided to allow replication by others?

Yes

Reviewer Expertise:

Consumer behaviour, services marketing

I confirm that I have read this submission and believe that I have an appropriate level of expertise to confirm that it is of an acceptable scientific standard.

# Reviewer report: Reviewer response for version 3

**DOI:** 10.5256/f1000research.207392.r509564

**Published:** 2026-08-10

**Authors:** The Anh Phan

**Affiliations:** 1 Eastern International University, Thu Dau Mot City, Vietnam

Dear Authors,

 I think fundamental problems involving construct validity, sampling, model specification, and theoretical inference prevent me from supporting indexing.

  

- Mandatory healthcare is not established as the study context. Compulsory enrolment in JKN does not mean that patients are compelled to use a particular facility. Participants also include both JKN-insured and general patients, yet their status is not reported or modelled. Consequently, the central boundary condition underlying the claimed contribution remains unobserved.

 

- Constrained choice is similarly assumed rather than measured. No item captures switching restrictions, perceived choice, administrative barriers, or dependence on the assigned facility. Claims concerning mandatory services and captive patients therefore extend beyond the data.

 

- Levels of analysis are misaligned. Omnichannel integration includes Mobile JKN, BPJS infrastructure, facility websites, WhatsApp, and in-person services operated by different actors. Brand trust and advocacy refer only to the healthcare facility. Patients may attribute ecosystem integration to BPJS or Mobile JKN rather than the focal facility. Current model does not resolve this attribution problem.

 

- Omnichannel integration lacks content validity. Items concerning promotions, contact information, listed services, self-check-in, and selection of service units primarily measure digital-feature availability. They do not necessarily measure consistency, continuity, synchronisation, or seamless movement across channels. Several descriptions in Table 2 also differ from the illustrative cross-channel items reported in Section 3.2.

 

- Reflective specification of this construct is not justified. Promotions, appointment functions, record access, complaint handling, and post-service support are distinct capabilities. They are not interchangeable manifestations of one latent perception. A multidimensional or formative specification may be more appropriate.

 

- Brand advocacy is incorrectly operationalised. BA1 measures willingness to try new programmes and services. BA2 measures positive word-of-mouth. BA3 measures forgiveness or willingness to return after disappointment. Only BA2 directly resembles advocacy.

 

- No item clearly measures recommendation or active brand defence, although both are repeatedly presented as dimensions of the scale. Calling BA3 “brand defence” does not change its actual wording. Authors later acknowledge that it may measure forgiveness, which confirms the construct-validity problem.
- Reported outcome is also described as behaviour despite being largely based on intentions and willingness. Statements that patients “engage in advocacy behaviours” are therefore unsupported. Statistical reliability cannot compensate for indicators representing different concepts.

 

- Brand trust may also be contaminated by service-quality evaluation. Perceived safety and reliability can reflect the physician encounter or clinical outcome rather than trust generated by channel integration. Because questionnaires were administered immediately after consultation, recent service experience is an especially plausible confounder. Service quality, satisfaction, health outcome, visit purpose, illness severity, waiting time, and prior relationship with the facility are omitted. Each could influence perceived integration, trust, and positive communication simultaneously. Demographic controls do little to address this omitted-variable problem.

 

- Pooling the three facilities is not defensible without institutional controls. Ownership, level of care, service quality, digital maturity, and patient composition may generate systematic differences in all three constructs. Between-facility differences could therefore produce the observed relationships. Facility dummy variables, within-facility estimates, and measurement-invariance tests are needed. Claims that implementation variation was established through researcher observation remain unverifiable. No results from the “structured availability check” are presented. Information reported in the author response suggests that all facilities offered similar channels, which does not demonstrate meaningful variation.

 

- Sampling procedures remain incomplete. Authors do not report how many patients were approached, how many refused, who administered the questionnaire, how privacy was protected during completion, or how incomplete and patterned responses were assessed. Reporting that all 230 questionnaires were valid provides no assurance of data quality.

 

- An “effective response rate” of 100% is incorrect when refusals were not recorded. Authors explicitly acknowledged this problem in their response to the previous reviewer, yet Version 3 still reports a 100% response rate. This discrepancy reduces confidence in the revision.

 

- Approximately equal facility samples and unusually balanced age, gender, and education distributions require explanation. If quotas were imposed, they must be disclosed. Describing the design only as purposive sampling is insufficient.

 

- Eligibility criteria create substantial selection bias. Every participant was a repeat user who had adopted a digital channel and completed an omnichannel journey. Digitally excluded patients, dissatisfied non-returning patients, first-time users, and those unable to navigate Mobile JKN are absent. Findings cannot be generalised to JKN patients or Indonesian healthcare users broadly. Power analysis is not reproducible. A minimum sample of 110 is reported without the assumed effect size, significance level, statistical power, number of predictors, calculation method, or software. Adequacy for a pooled model also does not resolve the facility-level dependence.

 

- Cross-sectional mediation cannot establish a transmission mechanism. Trust may shape perceptions of channel integration, while favourable patients may rate every construct positively. Temporal ordering among integration, trust, and advocacy is not observed.

 

- A significant indirect effect combined with a non-significant direct path does not prove full mediation. VAF is conditional on the selected model and cannot demonstrate that trust is the exclusive mechanism. Satisfaction, convenience, perceived control, service quality, risk reduction, and engagement were not modelled. Null direct effect is overinterpreted. Its confidence interval ranges from −0.119 to 0.157, which does not establish equivalence to zero. No equivalence test or smallest effect size of interest is provided. Phrases such as “entire association,” “will not directly stimulate,” and “insufficient to generate advocacy” should therefore be removed.

 

- Theoretical novelty remains modest. Positioning trust between a favourable service-system evaluation and a favourable behavioural response represents a conventional S-O-R mediation model. Commitment-trust theory is used mainly to justify one path, while service-dominant logic is not operationalised. No evidence demonstrates that this mechanism is distinctive to credence, high-risk, or mandatory services. Cross-context conclusions are unsupported. No retail, voluntary-service, low-risk, or experience-good comparison group is included. Comparing the magnitude of coefficients with unrelated published studies cannot establish that trust carries greater diagnostic weight in healthcare.

 

- Common-method assessment is unconvincing. Harman’s first factor explains 48.12%, which is close to the arbitrary 50% threshold and cannot rule out bias. Inter-construct correlations and inner VIF are not tests of common-method variance. Converting all items into positively worded statements may increase acquiescence rather than reduce it.

 

- Unmeasured latent method-factor analysis is not reproducible. No model diagram, method loadings, identification procedure, software output, or item-level results are provided. Reporting that the method factor explained 5.8% of variance is insufficient to evaluate the analysis.

 

- Instrument adaptation requires much greater transparency. Verhoef et al. provides conceptual omnichannel work rather than an obvious source for the reported healthcare scale. Extensive wording changes were made, including changes from retail to healthcare and from negative to positive formulations. A single expert and five patients cannot establish content validity after such substantial modification.
- Pre-test results are merely asserted. No loadings, reliability estimates, item revisions, deletion decisions, or participant characteristics are reported. Main-sample validity statistics do not retrospectively validate the adaptation process.

 

- PLS-SEM is not justified for this simple mediation model. Authors do not explain why a variance-based composite approach was preferred over covariance-based SEM. Reflective versus formative measurement assumptions, software, version, estimation settings, bootstrap procedure, missing-data treatment, and reproducible analysis code are also absent.
- Model comparison between the three-factor and merged two-factor solutions is not adequately reported. “Lower residual sum of squares” is presented without values, criteria, or testing procedures. Higher AVE after separating conceptually heterogeneous items is not a valid model-comparison test.
- Predictive claims are overstated. Blindfolding Q² is an in-sample predictive-relevance diagnostic, not evidence of out-of-sample prediction. PLSpredict, holdout validation, or another genuine predictive assessment is required before claiming predictive performance.
- Control-variable analysis is not transparently reported. Coefficients, standard errors, confidence intervals, coding schemes, and paths are absent. Facility, JKN status, channel type, visit frequency, and service experience would be more theoretically relevant than the reported demographic controls.
- Several serious inconsistencies remain despite the authors’ revision claims. Abstract attributes β = 0.671 to omnichannel integration and brand advocacy, although this coefficient represents brand trust to brand advocacy. Figure 1 still labels the stimulus as “Omnichannel Retailing,” despite the response stating that it had been replaced. Methods still report a 100% response rate despite the response stating that it could not be calculated.
- Figure 1 also treats H4 as if mediation were a separate structural path. Mediation arises from the indirect OCI–BT–BA sequence and should not be represented as an additional relationship. Model presentation should distinguish hypotheses from estimated paths.
- Reference integrity requires a complete audit. Reference 11 concerns service climate and employee customer-oriented behaviour rather than healthcare risk. Reference 16 concerns company-initiated engagement and shareholder value rather than cross-channel integration and trust. Reference 35 is listed as an international-marketing omnichannel study but is cited as evidence concerning quality cues in transformative healthcare systems. References 2 and 18 appear to duplicate the same article.
- Practical recommendations exceed the evidence. Referral programmes, digital testimonials, and patient advocacy initiatives were not tested. Encouraging patients to defend a facility or overlook disappointing experiences also raises patient-safety and ethical concerns that receive no discussion.
- The literature review should be further strengthened to better position the manuscript. In particular, I suggest incorporating and discussing the following recent studies, which may help deepen the theoretical framing and broaden the review’s relevance: "Selling health or communicating care? A bibliometric mapping of health marketing communications" "Live Influence: Redefining Credibility and Attractiveness in Healthcare Livestreaming" "Digital dilemmas: the complex interplay of online experience and health pharmaceutical purchases among Vietnamese women"
- Language and terminology remain inconsistent. Expressions such as “TB,” “OCH,” “path ratios,” and “advocacy makes an instrument of retention” require correction. Terms such as advocacy, recommendation, forgiveness, retention, and defence are used interchangeably despite representing different outcomes.

Is the work clearly and accurately presented and does it cite the current literature?

No

If applicable, is the statistical analysis and its interpretation appropriate?

Partly

Are all the source data underlying the results available to ensure full reproducibility?

No

Is the study design appropriate and is the work technically sound?

No

Are the conclusions drawn adequately supported by the results?

No

Are sufficient details of methods and analysis provided to allow replication by others?

Partly

Reviewer Expertise:

Health care Services

I confirm that I have read this submission and believe that I have an appropriate level of expertise to confirm that it is of an acceptable scientific standard, however I have significant reservations, as outlined above.

# Reviewer report: Reviewer response for version 2

**DOI:** 10.5256/f1000research.205853.r501330

**Published:** 2026-07-23

**Authors:** Nuria Viejo-Fernández

**Affiliations:** 1 University of Oviedo, Oviedo, Spain

I appreciate the authors' careful and constructive revision of the manuscript. They have addressed the substantive concerns raised in my previous review, particularly regarding the interpretation of causality, the description of the sampling procedure, the clarification of the reported response rate, the conceptualisation of omnichannel integration, the discussion of mediation, and the acknowledgement of the study's limitations. These revisions have substantially strengthened the manuscript.

 I only noted two minor issues that appear to remain in the revised version.

- In Table 7 and the corresponding text in Section 4.4, the 95% confidence interval for the non-significant direct path from omnichannel integration to brand advocacy is still reported as [0.119, 0.157]. Given β = 0.019, t = 0.263, and p = 0.793, the lower bound should be negative. The authors’ response also confirms that the correct interval is [−0.119, 0.157]. This minus sign should therefore be restored in both the table and the main text.
- I also recommend replacing “Omnichannel Retailing” with “Omnichannel Integration” in Figure 1 so that the terminology in the conceptual model is consistent with the construct used throughout the revised manuscript.

Subject to these two straightforward corrections, particularly the correction of the confidence interval, I would be satisfied that my previous reservations have been adequately addressed.

Is the work clearly and accurately presented and does it cite the current literature?

Yes

If applicable, is the statistical analysis and its interpretation appropriate?

Yes

Are all the source data underlying the results available to ensure full reproducibility?

Yes

Is the study design appropriate and is the work technically sound?

Partly

Are the conclusions drawn adequately supported by the results?

Partly

Are sufficient details of methods and analysis provided to allow replication by others?

Partly

Reviewer Expertise:

Marketing, consumer behaviour, omnichannel retailing, digital transormation.

I confirm that I have read this submission and believe that I have an appropriate level of expertise to confirm that it is of an acceptable scientific standard, however I have significant reservations, as outlined above.

# Response

**Published:** 2026-07-27

**Authors:** Erwin Suryanegara

**Affiliations:** Management, Universitas Esa Unggul, West Jakarta, Jakarta, Indonesia

We thank the reviewer for the careful re-reading of the manuscript and for confirming that the substantive concerns raised in the first round have been addressed. We have now made both remaining corrections, and a revised version has been submitted to the journal.

 First, the negative sign on the lower bound of the 95% confidence interval for the direct path from omnichannel integration to brand advocacy has been restored in both Table 7 and Section 4.4, which now read [-0.119, 0.157]. We note that the minus sign was present in the file we submitted but was encoded as a Unicode minus (U+2212) and was lost during typesetting; it has been re-entered as a standard hyphen-minus, and we have asked the production team to verify that it renders correctly in the proof.

 Second, Figure 1 has been replaced so that the stimulus construct is labelled "Omnichannel Integration" rather than "Omnichannel Retailing", making the conceptual model terminologically consistent with the construct used throughout the manuscript.

 No data, analyses, results, or conclusions have changed. We are grateful for the reviewer's constructive engagement, which has improved the clarity and accuracy of the article.

# Reviewer report: Reviewer response for version 1

**DOI:** 10.5256/f1000research.202357.r495159

**Published:** 2026-06-30

**Authors:** Nuria Viejo-Fernández

**Affiliations:** 1 University of Oviedo, Oviedo, Spain

The article examines whether brand trust mediates the relationship between perceived omnichannel integration and brand advocacy in mandatory healthcare services in Indonesia. Drawing on the S-O-R framework, the authors analyse survey data from 230 patients from three healthcare facilities in Jakarta and Tangerang using PLS-SEM. The results show that omnichannel integration is positively associated with brand trust, and that brand trust is positively associated with brand advocacy. The direct effect of omnichannel integration on advocacy becomes non-significant once trust is included, leading the authors to interpret the relationship as full mediation.

 The study addresses a relevant and timely topic. The application of omnichannel integration to mandatory healthcare services is interesting, and the focus on trust as a psychological mechanism is theoretically appropriate for credence services where patients face high perceived risk and limited ability to evaluate service quality directly. The manuscript is generally clearly written, the literature review is coherent, and the authors make a useful effort to define the meaning of “brand” in this specific healthcare context.

 However, I have some reservations that should be addressed to strengthen the scientific soundness of the article.

 First, the study design is appropriate for testing associations, but the cross-sectional nature of the data does not allow strong causal claims. The language in some parts of the manuscript should be softened. The authors should consistently refer to “associations” rather than “effects” unless they clearly justify the causal interpretation. This is particularly important for the mediation analysis, since mediation tested with cross-sectional survey data cannot demonstrate temporal ordering.

 Second, more detail is needed on the sampling procedure. The authors state that purposive sampling was used and that all 230 questionnaires were valid, resulting in a 100% effective response rate. This requires clarification. The manuscript should report how patients were approached, how many refused to participate, whether any questionnaires were excluded before analysis, and whether the final sample may over-represent more digitally literate or more satisfied patients.

 Third, the operationalisation of omnichannel integration would benefit from further clarification. Some items seem to capture the availability of online information or digital services rather than true integration across channels. The authors should explain more clearly how the selected indicators reflect integration, continuity, and consistency rather than simple multichannel access.

 Fourth, the interpretation of “full mediation” should be more cautious. The statistical results support a non-significant direct path and a significant indirect path, but the authors should avoid overstating this as proof that omnichannel integration only works through trust. Alternative mechanisms, such as satisfaction, perceived service quality, perceived control, convenience, or perceived risk reduction, are not included in the model and may also explain advocacy.

 Fifth, the three facilities differ in ownership type, level of care, and likely degree of digital maturity. Although this variation is useful, the manuscript would be stronger if the authors provided descriptive information by facility or tested whether the structural relationships are stable across facility types. At minimum, this issue should be acknowledged as a limitation.

 The statistical analysis is generally appropriate. The authors report reliability, convergent validity, discriminant validity, HTMT, common method bias checks, bootstrapping, mediation, and control variables. These procedures are suitable for the proposed PLS-SEM approach. Nevertheless, the authors should ensure that all reported confidence intervals are correctly presented, especially where the lower bound appears to be missing a negative sign for the non-significant direct path.

 The source data are reported as openly available, which supports reproducibility. However, the authors should make sure that the dataset includes clear variable labels, item wording, coding information, and sufficient documentation for another researcher to reproduce the analysis.

 In conclusion, the article makes a useful contribution to omnichannel service research by extending the discussion to mandatory healthcare and by highlighting the central role of trust. I consider the work promising and technically acceptable, but I recommend that the authors revise the manuscript to address the methodological clarifications above, especially the limits of causal interpretation, the sampling procedure, and the measurement of omnichannel integration.

Is the work clearly and accurately presented and does it cite the current literature?

Yes

If applicable, is the statistical analysis and its interpretation appropriate?

Yes

Are all the source data underlying the results available to ensure full reproducibility?

Yes

Is the study design appropriate and is the work technically sound?

Partly

Are the conclusions drawn adequately supported by the results?

Partly

Are sufficient details of methods and analysis provided to allow replication by others?

Partly

Reviewer Expertise:

Marketing, consumer behaviour, omnichannel retailing, digital transormation.

I confirm that I have read this submission and believe that I have an appropriate level of expertise to confirm that it is of an acceptable scientific standard, however I have significant reservations, as outlined above.

# Response

**Published:** 2026-06-30

**Authors:** Erwin Suryanegara

**Affiliations:** Management, Universitas Esa Unggul, West Jakarta, Jakarta, Indonesia

Dear Dr. Viejo-Fernández,

 Thank you very much for your thorough and constructive review, and for approving our article. We greatly appreciate your insightful comments, which will help us strengthen the scientific soundness of the manuscript. We will address each point in the revised version as follows:

1. **Causal interpretation.** We agree that the cross-sectional design does not support strong causal claims. In the revision we will consistently use the term "associations" rather than "effects," and we will explicitly acknowledge in the limitations section that the mediation results indicate statistical association rather than temporal/causal ordering.
2. **Sampling procedure.** We will expand the methods section to describe how patients were approached, the number who declined to participate, any questionnaires excluded prior to analysis, and we will discuss the possibility that the final sample may over-represent more digitally literate or more satisfied patients.
3. **Operationalisation of omnichannel integration.** We will clarify how the selected indicators capture integration, continuity, and consistency across channels rather than merely the availability of online services, and we will acknowledge this as a limitation where relevant.
4. **Statistical analysis.** Thank you for catching this. We will carefully re-check all reported confidence intervals, including the lower bound of the non-significant direct path, and correct any missing negative sign.
5. **Open data.** We will ensure the deposited dataset includes clear variable labels, item wording, and coding information, with sufficient documentation to allow another researcher to reproduce the analysis.

We are grateful for your valuable feedback and believe these revisions will substantially improve the paper. We will submit a revised version addressing all of the above.

 Best regards,

 Erwin Suryanegara

 On behalf of all authors

# Response

**Published:** 2026-07-08

**Authors:** Erwin Suryanegara

**Affiliations:** Management, Universitas Esa Unggul, West Jakarta, Jakarta, Indonesia

We thank the reviewer for the thorough and constructive report. We have revised the manuscript to address each reservation.

**Comment 1 (Cross-sectional data — soften causal language).** Revised throughout. The Abstract now closes with: "As the data are cross-sectional, the reported relationships represent associations rather than causal effects." Interpretive claims in Sections 4.5 and 5.3 have been softened ("consistent with full mediation," "within the tested model," "may not directly stimulate"), and the first limitation now states explicitly that mediation tested with cross-sectional data cannot demonstrate temporal ordering, so the full-mediation pattern is consistent with—rather than demonstrative of—the hypothesised sequence. We retained the standard statistical terms "direct effect" and "indirect effect" in reporting the PLS-SEM results, as these are conventional path-analytic labels rather than causal claims. We also corrected a path-attribution error in the Abstract: β = 0.671 is now correctly attributed to the brand trust → brand advocacy path.

**Comment 2 (Sampling procedure and the 100% response rate).** Section 3.1 now reports that patients were approached by nurses immediately after the physician consultation and questionnaires were administered only to those who agreed; no questionnaires were excluded before analysis. We clarify that the 100% figure is a usable-questionnaire rate reflecting supervised, in-person administration; because refusals at recruitment were not recorded, a conventional response rate cannot be computed, and the manuscript now says so explicitly. The Limitations additionally acknowledge that the sample may over-represent more digitally literate or more favourably disposed patients.

**Comment 3 (Operationalisation of omnichannel integration).** Section 3.2 now explains that the OCI indicators capture the perceived quality of integration—consistency of information across channels, continuity of medical and administrative records when moving between channels, and support experienced when switching channels—dimensions that presuppose, but go beyond, simple multichannel access. Channel availability itself is documented separately at the facility level (Section 3.1).

**Comment 4 (Interpretation of full mediation).** Section 5.3 now states that trust was the only mediator specified and that alternative mechanisms—satisfaction, perceived service quality, perceived control, convenience, or perceived risk reduction—were not modelled and may also transmit part of the omnichannel–advocacy association; the full-mediation pattern is presented as evidence of trust's centrality within the tested specification rather than proof of an exclusive pathway.

**Comment 5 (Facility heterogeneity).** Section 3.1 describes the three facilities' ownership types, levels of care, patient segments, and channel environments, with approximately equal subsamples (n = 77/77/76). We did not conduct a multi-group analysis given the per-facility sample sizes, and this is acknowledged explicitly in the Limitations as recommended.

**Comment 6 (Confidence interval sign).** Thank you for catching this. In the revised manuscript, the 95% CI for the non-significant direct path correctly reads [−0.119, 0.157] (Table 7 and Section 4.4); the minus sign appears to have been lost during typesetting of Version 1, and we will check the Version 2 proofs carefully.

**Comment 7 (Dataset documentation).** The figshare record accompanying the article has been updated to Version 2 (DOI: 10.6084/m9.figshare.32356992.v2) to include a codebook file documenting variable names, full item wording, and response coding sufficient to reproduce the analysis.

# Reviewer report: Reviewer response for version 1

**DOI:** 10.5256/f1000research.202357.r495151

**Published:** 2026-06-25

**Authors:** Sudhinder Singh Chowhan

**Affiliations:** 1 IIHMR University, Jaipur, India

**1. Abstract**

**Minor correction**

 The statement: "The model explained 46.8% of the variance in brand advocacy." should be explicitly linked to the PLS-SEM model.

 Suggested revision: "The structural model explained 46.8% of the variance in brand advocacy (R² = 0.468)."

**2. Introduction**

**Clarify theoretical novelty**

 The manuscript repeatedly states that healthcare differs from retail because of credence characteristics, high perceived risk and constrained choice.

 A concise paragraph synthesizing these three characteristics into one theoretical proposition would strengthen the introduction.

 Suggested addition: "Taken together, these characteristics suggest that omnichannel integration may influence patient behavior less through experiential value and more through trust formation, making healthcare a theoretically distinct setting for examining omnichannel mechanisms."

**3. Literature Review**

 H3 hypothesizes a direct OCI → BA effect.

 Although trust is expected to explain an important portion of the relationship, omnichannel integration may also create direct experiential benefits such as convenience and reduced effort, which can independently stimulate advocacy.

**4. Methods**

**Sampling justification**

 The manuscript reports "researcher observation and structured availability check" to establish variation in omnichannel implementation.

 Consider adding a brief appendix or supplementary table showing Mobile JKN integration, Website availability, WhatsApp services, Online appointment system, and electronic records access for each facility. This would improve reproducibility.

**5. Measurement Development**

**Brand Advocacy construct**

 The limitation section already acknowledges potential concerns regarding BA3.

 Consider flagging this earlier in the measurement section. BA3 reflects a defensive form of advocacy and was retained because advocacy literature conceptualizes defense as a higher-order advocacy behavior. This pre-empts reviewer concerns.

**Overall Referee Recommendation**

 Decision: **Approved** with Minor Revisions

 Strengths include:

- Strong theoretical grounding in S-O-R theory.
- Well-developed healthcare-specific omnichannel conceptualization.
- Appropriate use of PLS-SEM.
- Extensive validity and robustness checks.
- Transparent reporting of mediation analysis.
- Open data and instrument availability.

The recommended revisions are largely editorial and interpretive, aimed at improving transparency, reproducibility, and consistency between evidence and conclusions rather than requiring additional analyses.

Is the work clearly and accurately presented and does it cite the current literature?

Yes

If applicable, is the statistical analysis and its interpretation appropriate?

Yes

Are all the source data underlying the results available to ensure full reproducibility?

Yes

Is the study design appropriate and is the work technically sound?

Yes

Are the conclusions drawn adequately supported by the results?

Yes

Are sufficient details of methods and analysis provided to allow replication by others?

Yes

Reviewer Expertise:

Pharmaceutical Marketing

I confirm that I have read this submission and believe that I have an appropriate level of expertise to confirm that it is of an acceptable scientific standard.

# Response

**Published:** 2026-06-25

**Authors:** Erwin Suryanegara

**Affiliations:** Management, Universitas Esa Unggul, West Jakarta, Jakarta, Indonesia

Dear Dr. Chowhan,

 Thank you very much for your constructive review and for approving our article. We sincerely appreciate your helpful suggestions regarding the abstract, theoretical novelty, sampling justification, and measurement clarification.

 We acknowledge these minor points and will consider them carefully when preparing a revised version of the article after receiving any additional reviewer reports, in line with the editorial recommendation.

 Thank you again for your valuable feedback.

 Best regards,

 Erwin Suryanegara

 On behalf of all authors

# Response

**Published:** 2026-07-08

**Authors:** Erwin Suryanegara

**Affiliations:** Management, Universitas Esa Unggul, West Jakarta, Jakarta, Indonesia

We thank the reviewer for the careful reading and the constructive, actionable suggestions. All recommended revisions have been implemented.

**Comment 1 (Abstract — link variance to the PLS-SEM model).** Revised as suggested. The Abstract now reads: "the structural model explained 46.8% of the variance in brand advocacy (R² = 0.468)."

**Comment 2 (Introduction — synthesise the three characteristics into one theoretical proposition).** Added as suggested. The final paragraph of the Introduction now states that, taken together, credence attributes, constrained choice, and elevated perceived risk suggest that omnichannel integration may influence patient behaviour less through experiential value and more through trust formation, making health care a theoretically distinct setting for examining omnichannel mechanisms.

**Comment 3 (H3 — direct experiential benefits).** We agree. The rationale for H3 (Section 2.4) now explicitly states that omnichannel integration provides intrinsic experiential value—access convenience, reduced effort, and service personalisation—that may independently stimulate advocacy, coexisting with the trust pathway.

**Comment 4 (Sampling — document omnichannel implementation per facility).** Implemented in narrative form in Section 3.1, which now documents the structured availability check for each facility: Mobile JKN integration, facility website, WhatsApp service, online appointment booking, and walk-in service were available at all three sites, while patient-facing access to electronic medical records was partial at all sites (visit history viewable through Mobile JKN; full records not directly accessible). We integrated this into Section 3.1 rather than an appendix table so the information sits alongside the facility descriptions; we are happy to convert it to a supplementary table if preferred.

**Comment 5 (Measurement — flag BA3 earlier).** Done. Section 3.2 now flags at the measurement stage that BA3 captures a give-another-chance dimension of advocacy, retained because the advocacy literature treats defence of and continued commitment to a provider as advocacy behaviour, with a forward reference to the Limitations. For consistency, the characterisation of BA3 has also been aligned across Sections 3.2, 4.1 (descriptive statistics), 5.2, and the Limitations.
